# Fast DOA Estimation Algorithms via Positive Incremental Modified Cholesky Decomposition for Augmented Coprime Array Sensors

**DOI:** 10.3390/s23218990

**Published:** 2023-11-05

**Authors:** Jing Song, Lin Cao, Zongmin Zhao, Dongfeng Wang, Chong Fu

**Affiliations:** 1 School of Artificial Intelligence, China University of Mining and Technology (Beijing), Beijing 100083, China; sungking@student.cumtb.edu.cn; 2 Key Laboratory of the Ministry of Education for Optoelectronic Measurement Technology and Instrument, Beijing Information Science and Technology University, Beijing 100101, China; zhaozongmin@bistu.edu.cn; 3 School of Information and Communication Engineering, Beijing Information Science and Technology University, Beijing 100101, China; 4 Beijing TransMicrowave Technology Company, Beijing 100080, China; wdf@tsmtc.com; 5 School of Computer Science and Engineering, Northeastern University, Shenyang 110169, China; fuchong@mail.neu.edu.cn

**Keywords:** DOA estimation, virtual interpolation, covariance matrix reconstruction, atomic norm minimization, positive incremental modified Cholesky decomposition

## Abstract

This paper proposes a fast direction of arrival (DOA) estimation method based on positive incremental modified Cholesky decomposition atomic norm minimization (PI-CANM) for augmented coprime array sensors. The approach incorporates coprime sampling on the augmented array to generate a non-uniform, discontinuous virtual array. It then utilizes interpolation to convert this into a uniform, continuous virtual array. Based on this, the problem of DOA estimation is equivalently formulated as a gridless optimization problem, which is solved via atomic norm minimization to reconstruct a Hermitian Toeplitz covariance matrix. Furthermore, by positive incremental modified Cholesky decomposition, the covariance matrix is transformed from positive semi-definite to positive definite, which simplifies the constraint of optimization problem and reduces the complexity of the solution. Finally, the Multiple Signal Classification method is utilized to carry out statistical signal processing on the reconstructed covariance matrix, yielding initial DOA angle estimates. Experimental outcomes highlight that the PI-CANM algorithm surpasses other algorithms in estimation accuracy, demonstrating stability in difficult circumstances such as low signal-to-noise ratios and limited snapshots. Additionally, it boasts an impressive computational speed. This method enhances both the accuracy and computational efficiency of DOA estimation, showing potential for broad applicability.

## 1. Introduction

Direction of arrival (DOA) estimation is a critical area of study within array signal processing, impacting fields such as radar, communications, sonar, and exploration [1,2,3,4]. Notably, array-based spatial spectrum estimation techniques such as multiple signal classification (MUSIC) and estimation of signal parameters via rotational invariance techniques (ESPRIT) have become the go-to methods for DOA estimation. Several studies have been conducted on these techniques. For instance, the statistical characteristics of the MUSIC algorithm were investigated in one study [5], while another study used the low-complexity GA-ESPRIT algorithm for DOA estimation [6]. Furthermore, a comprehensive study on the application of MUSIC and ESPRIT in designing intelligent antenna systems revealed that MUSIC outperforms ESPRIT in precision, stability, and broader application [7]. These subspace-based methods have dramatically improved direction-finding and localization technologies by surpassing the Rayleigh limit. However, they were primarily designed for traditional uniform linear arrays (ULAs). Enhancing estimation accuracy and resolution necessitates an increase in the array elements, thereby escalating the algorithmic complexity. This complexity is particularly pronounced in underdetermined DOA estimation, where performance can drastically decrease. Moreover, these methods necessitate extensive independently and identically distributed observational data. In real-world environments, especially when tracking fast-moving targets, acquiring such data can be a time-consuming task [8,9]. Dealing with short-duration bursty data becomes a challenge as the received signals contain an extremely limited number of sample snapshots. This limitation impedes traditional DOA estimation methods from accurately locating the target. To overcome these hurdles, some researchers have turned to novel solutions, such as compressed sensing methods. By leveraging signal sparsity, these methods compress measurement data with fewer sampling points and accurately recover the original signal [10]. As a result, these approaches provide a new perspective for precise DOA estimation when the data volume is constrained.

Traditional ULAs necessitate half-wavelength spacing between adjacent elements, often leading to coupling effects. Moreover, the limited array aperture restricts the precision and resolution of DOA estimation. To address this issue, researchers introduced a sparse array known as a coprime array. It leverages the covariance matrix to construct a difference co-array in the virtual domain, resulting in a virtual array with a greater number of elements [11]. Several studies have examined DOA estimation methods using coprime array sensors from varying viewpoints. These include subarray decomposition, subspace decomposition in the virtual domain, and sparse reconstruction. References [12,13] applied the MUSIC algorithm directly for DOA estimation in coprime array sensors. Meanwhile, reference [14] proposed a DOA estimation method suitable for correlated signals in coprime array sensors. Lastly, reference [15] presented a DOA estimation strategy based on coprime array sensors in an unknown non-uniform noise environment. The DOA estimation method of the coprime array sensors mentioned earlier takes advantage of the large aperture of the array. However, these methods only utilize consecutive elements in the virtual array, resulting in information loss. To exploit virtual array elements more thoroughly, some researchers have developed sparse reconstruction problems in the virtual domain, aiming to obtain more accurate DOA estimates. Reference [11] introduced a sparse sensing approach for coprime sampling and arrays. Reference [16] suggests a least absolute shrinkage and selection operator (CO-LASSO) algorithm focused on sparse signal recovery for DOA estimation. This method employs an overcomplete basis representation of the spatial power spectrum and constructs a data fitting model. Reference [17] presented a sparse Bayesian learning (SBL) approach for robust DOA estimation. It establishes a Bayesian model that integrates prior information and observational data utilizing Bayesian inference techniques for iterative optimization. Other noteworthy methods include a DOA estimation technique based on a padding strategy [18] and a source estimation method grounded in sparse reconstruction [19]. The previously discussed sparse reconstruction methods efficiently use the benefits of the coprime array sensors’ virtual array aperture and degrees of freedom, enabling a relatively precise DOA estimation. However, they require partitioning the continuous angular space into discrete grids and also presuppose the source direction lies precisely within a predetermined discrete grid. These assumptions result in a basic mismatch problem, which in turn lowers the algorithm’s performance. Another class of virtual domain subspace methods achieves DOA estimation by incorporating spatial smoothing techniques, developing a signal model, and restoring the covariance matrix’s rank within the coprime array sensors’ virtual domain [20,21]. In contrast to coprime array sensors’ sparse reconstruction methods, this strategy avoids the requirement of angular domain discretization, thereby successfully reducing model mismatch issues. Nevertheless, these methods do not completely exploit the components of the virtual array, leading to a partial loss of virtual aperture and degrees of freedom. This, in turn, can cause a decrease in the performance of the DOA estimation.

In recent years, with the development of compressed sensing technology, DOA estimation based on covariance matrix reconstruction and completion has become a research hotspot [22,23]. This approach firstly entails the reconstruction of the covariance matrix, which improves the aperture and degrees of freedom. Following this, methods such as singular value thresholding are utilized to fill the gaps in the reconstruction, culminating in the final angle estimation. Zheng et al. proposed an augmented covariance matrix reconstruction method utilizing differential co-arrays [24]. Shortly thereafter, Barthelme et al. introduced a covariance matrix reconstruction method for estimation based on neural networks [25]. Also, research have focused on DOA estimation using structured methods such as virtual array interpolation and reciprocal arrays. In this regard, the work of Zhou et al. is very representative, they proposed a method based on structured Nyquist correlation reconstruction [26]. This method is applicable to DOA estimation in sparse arrays, exhibiting high estimation accuracy. Furthermore, gridless compressive sensing methods based on atomic norm minimization (ANM) have made some significant breakthroughs. For instance, research by Li et al. [27] has viewed ANM as a structural optimization approach, which by leveraging the Vandermonde structure of the array manifold matrix, acquires the necessary signal covariance matrix through solving semi-definite programming (SDP) problems. Zhou et al. [28] combined the ANM method with the MUSIC algorithm for DOA estimation on co-prime arrays, effectively resolving basis mismatch issues and enhancing the estimation precision. However, these methods encounter slow solving speeds when utilizing convex optimization toolboxes to tackle optimization problems within sparse recovery algorithms, making them unsuitable for larger-scale array systems. To address this problem, Lau et al. [29] introduced a low-complexity single-snapshot angle estimation method that merges prior information, achieving a higher degree of atomic separation freedom. Wang et al. [30] proposed a fast algorithm named iterative Vandermonde decomposition and shrinkage thresholding, which attains near-proximal gradient acceleration for ANM while avoiding computationally demanding SDP methods. Additionally, Ma et al. [31] presented a method to efficiently resolve SDP issues using a projection Wirtinger gradient descent algorithm. Therefore, the challenge of reducing the complexity of gridless methods such as ANM has emerged as a significant and worthy research topic.

**Figure 1:**
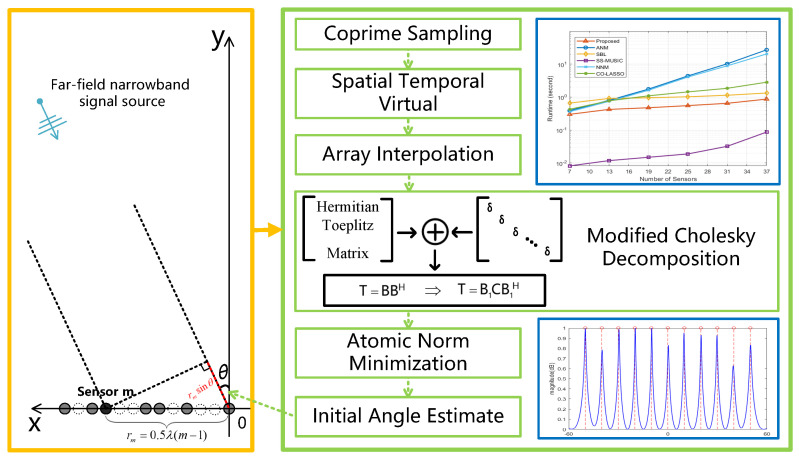
Overview of algorithm application scenarios, framework structure, and simulation results.

In this paper, a fast DOA estimation algorithm based on positive incremental modified Cholesky decomposition atomic norm minimization(PI-MCANM) is proposed, which is designed on the basis of sparse array antenna radar sensors. Figure 1 shows the application scenario of the algorithm, the framework, and some experimental simulation results. The algorithm defines the atomic norm of the received signal to establish a continuous set of atoms, converting the sparse reconstruction problem of the signal covariance matrix into a SDP optimization problem, which does not require grid division, thus avoiding the grid mismatch problem. To utilize all the information of the received signal from the augmented co-prime array, we use virtual interpolation technology to interpolate the original co-prime array difference into a continuous uniform array. In the process of solving the SDP problem, we also use the positive incremental modified Cholesky decomposition to preprocess the Toeplitz matrix, converting the original SDP problem into a new optimization problem, thereby reducing the complexity of the algorithm and achieving accurate and fast DOA estimation. Simulation results show the advantages of the proposed DOA estimation method in terms of resolution, estimation accuracy, and computational complexity.

The main contributions of this paper can be summarized as follows: (1) A novel augmented coprime array sensor DOA estimation algorithm is introduced using virtual interpolation technology to address information loss in non-uniform virtual differential arrays, fully utilizing received information; (2) The algorithm applies multiple atomic norms of virtual measurements to convert the DOA estimation issue into a gridless optimization problem, addressing the base mismatch issue from predefined spatial grid points; (3) The method employs positive incremental modified Cholesky decomposition to preprocess the covariance matrix, simplifying the optimization problem’s constraints and reducing its complexity.

The remainder of the paper reads as follows. Section 2 introduces the augmented coprime array sensors structure and its corresponding signal reception model. In Section 3, the proposed algorithm based on PI-MCANM is provided. Section 4 demonstrates the effectiveness and superiority of the proposed method through simulation experiments from multiple perspectives. Finally, we draw conclusions in Section 5.

## 2. Signal Model

### 2.1. Array Structure

Due to the Nyquist sampling theorem’s constraints on the uniform linear array, the original signal can only be effectively restored if the sampling rate exceeds twice the incident signal’s highest frequency. Therefore, the spatial separation between the linear array elements must not surpass half the wavelength of the incident signal, as expressed by the equation d⩽λ/2 [32]. Recently proposed, the coprime array sensors stand as a unique sparse array, overcoming the Nyquist sampling rate’s limitations. This array possesses the benefits of sparse perception and the special attributes of prime numbers. It can maximize the array’s aperture and degrees of freedom while maintaining the same count of physical array elements. Furthermore, the augmented coprime array sensors, as suggested in [33], create more virtual array elements in the same orientation and longer continuous virtual array elements than the traditional coprime array sensors. This feature can aid in applying subsequent spatial smoothing algorithms and other strategies, making it a valuable research direction for studying DOA estimation based on augmented coprime array sensors.

**Figure 2:**
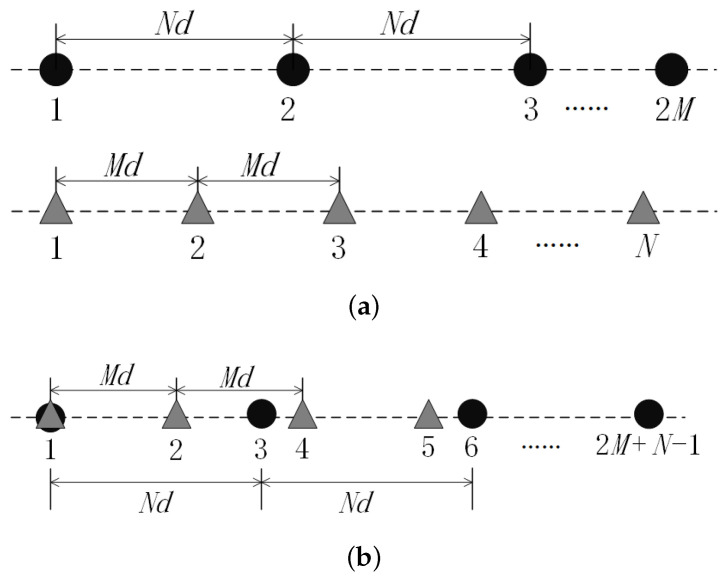
Schematic diagram of augmented coprime array structure. (**a**) Two uniform linear subarrays. (**b**) Augmented coprime array composed of two sparse uniform linear arrays.

This paper investigates augmented coprime array sensors, as mentioned in [13]. This array comprises two subarrays; both are uniform line arrays. The first subarray consists of 2M array elements spaced Nd apart. The second subarray has *N* array elements with a spacing of Md. Here, *M* and *N* are mutually prime integers, with *M* being the smaller of the two. It is assumed that *d* is the half-wavelength of the target signal that encounters the augmented coprime array sensors. The structure of this setup is demonstrated in Figure 2a. The augmented coprime array sensor is constructed by overlapping the initial array elements of the two subarrays and linearly superimposing them. This results in an array with a total of 2M+N−1 physical array elements. Figure 2b illustrates this structure.

### 2.2. Signal Reception Model

Assuming there is a set of unrelated signal sources received by sensors equipped with an augmented coprime array sensor system from different incident angles $\theta =\left[\theta_{1},\theta_{2},\cdots ,\theta_{K}\right]$ at time t, the received signals of the coprime array can be represented as

(1) X(t)=A(θ)S(t)+N(t),

where $A\left(\theta \right)={\left[a\left(\theta_{1}\right),a\left(\theta_{2}\right),\ldots ,a\left(\theta_{K}\right)\right]}^{T}$ represents the P×K-dimensional coprime array manifold matrix, where P=2M+N−1 is the number of physical elements in the augmented coprime array. Here, $a\left(\theta_{k}\right)={\left[1,e^{-j2\pi d_{1}\sin\theta_{k}/\lambda },\ldots ,e^{-j2\pi d_{p-1}\sin\theta_{k}/\lambda }\right]}^{T}$ denotes the array steering vector corresponding to the *k*-th incident signal, with the first element position set at 0. $d_{p-1}$ denotes the *p*th physical array element position in the coprime array. $S\left(t\right)={\left[s_{1}\left(t\right),s_{2}\left(t\right),\ldots ,s_{K}\left(t\right)\right]}^{T}$ denotes the complex envelope of the signals, and N(t) represents Gaussian white noise with the power $\sigma_{N}^{2}$, which is independent of the signal sources. The superscript T represents the transpose of the matrix. The covariance matrix computation based on the coprime array receiving signal model can be formulated as

(2) $$\begin{array}{cc} R & =E\left[X\left(t\right)X{\left(t\right)}^{H}\right]\\ \; & =E\left[\left(A\left(\theta \right)S\left(t\right)+N\left(t\right)\right){\left(A\left(\theta \right)S\left(t\right)+N\left(t\right)\right)}^{H}\right]\\ \; & =A\left(\theta \right)R_{s}A^{H}\left(\theta \right)+\sigma_{N}^{2}I \end{array},$$

where $R_{S}=E\left\{S\left(t\right)S{\left(t\right)}^{H}\right\}=\mathrm{diag}\left(\left[\sigma_{1}^{2},\sigma_{2}^{2},\cdots ,\sigma_{K}^{2}\right]\right)$ represents a K×K diagonal matrix, where the diagonal elements contain the power of each incident signal. *I* is an identity matrix with size *P* by *P*. E(·) denotes the statistical expectation operation, and ${\left(\cdot \right)}^{H}$ represents the conjugate transpose. Due to the limited number of samples in practice, the covariance matrix is typically estimated using *L* samples of received data, approximating it as

(3) $$R_{X}=\frac{1}{L}\sum_{l=1}^{L}X\left(t_{l}\right)X^{H}\left(t_{l}\right).$$

## 3. PI-MCANM-Based DOA Estimation Algorithm

This section introduces a fast DOA estimation method based on PI-MCANM for augmented coprime array sensors, including virtual interpolation techniques, atomic norm minimization methods, and the fast DOA estimation algorithm using positive incremental modified Cholesky decomposition. Firstly, virtual interpolation techniques are employed to fully exploit the spatial correlation within the array, allowing for the reconstruction of missing data in the virtual array. This effectively enhances the degrees of freedom and accuracy of the array. Secondly, the method utilizes atomic norm minimization for the reconstruction of the covariance matrix. By leveraging the sparsity of the signals, it enables the acquisition of crucial signal information at a lower sampling rate. Furthermore, while ensuring reconstruction accuracy, the approach combines sparse signal recovery techniques, such as non-negative matrix factorization, to seek the optimal solution and avoid the issue of local optima that may arise in traditional optimization algorithms. Finally, the DOA estimation algorithm combines the modified Cholesky decomposition preprocessing with atomic norm minimization to reconstruct the covariance matrix, thereby facilitating DOA estimation and improving the computational efficiency of the algorithm.

### 3.1. Virtual Array Interpolation

From Part A of Section 2, we know that the position set S of 2M+N−1 array elements in a coprime array can be expressed as

**Figure 3:**
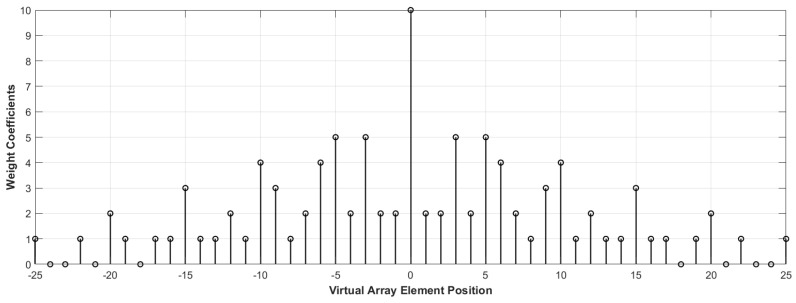
Weight coefficients corresponding to virtual array elements at different positions.

(4) S={Mnd,0≤n≤N−1}∪{Nmd,0≤m≤2M−1},

we can obtain the element positions of the difference coarry by taking the differences between any two elements in the augmented coprime array element set. The difference coarray is also referred to as the virtual array. Figure 3 shows the different positions of the array elements in the virtual array and the corresponding weight coefficients (i.e., the number of array elements) after the difference operation. It can be observed that the differencing operation increases the degrees of freedom of the original array. However, it introduces the issue of discontinuity in the differencing set, which can lead to the loss of information from certain array elements during DOA estimation. These non-continuous regions in the virtual array, where the weighting coefficients are zero, are referred to as holes. Based on the idea of the difference coarray, the set of differencing array positions D obtained by performing differencing operations on the elements in S can be represented as

(5) D={±(Mn−Nm)d;0≤n≤N−1,0≤m≤M−1},

by setting the number of array elements in this way, the location difference set of the augmented coprime array is continuous in the range of [−(MN+M−1)d,(MN+M−1)d]. Combining the knowledge of virtual array construction, we have constructed a virtual uniform linear array (ULA) with 2MN+2M−1 array elements using 2M+N−1 physical array elements. Taking the example of an augmented coprime array with M=3 and N=5, the position range of virtual differential array elements that can be constructed is from −25d to 25d, and the continuous part U is [−17d,17d]. Other positions with missing elements are called holes, located at {−24d,−23d,−21d,−18d,18d,21d,23d,24d}.

**Figure 4:**
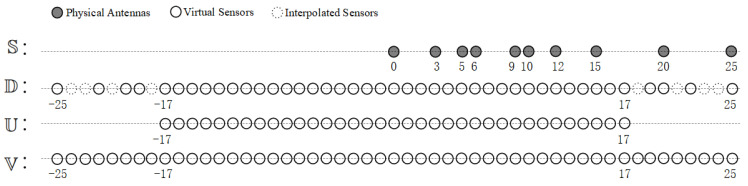
Illustration of various array representations with an example of 2*M* = 6 and *N* = 5. S represents the augmented coprime array. D is the virtual array derived from the difference coarray of the augmented coprime array. U represents the contiguous part of the virtual array. V is the interpolated virtual array.

To address the issue of reduced degrees of freedom caused by holes in the virtual difference array, we introduce the concept of interpolation in the virtual difference array. Considering that it is not possible to obtain the second-order equivalent virtual signals of the interpolated virtual array in practical signal processing, we treat the hole positions as inactive antenna elements. By filling the hole positions in the non-uniform virtual array D with zeros, we transform the non-uniform virtual array into a continuous virtual uniform linear array V containing 4M(N−1)+1 virtual elements, where the virtual uniform linear arrays are all spaced by *d*, i.e., half wavelengths. This approach resolves the model mismatch issue arising from the non-uniform structure. Figure 4 illustrates the actual element positions of the augmented coprime array and the constructed virtual element positions.

### 3.2. Atomic Norm Minimization

Atomic norm minimization is a method grounded in convex optimization, enabling gridless DOA estimation through the sparse representation of DOA angles. In essence, atomic norm minimization operates on the assumption that a finite number of atoms can form the sparse representation of DOA angles, with each atom corresponding to a potential DOA angle. The solution with the highest degree of sparsity can be obtained by solving a convex optimization problem, yielding precise DOA estimation results. The first step of this method is defining the atomic set

(6) $$A=\left\{a\left(\theta ,\phi \right)=a\left(\theta \right)\phi :\theta \in \left\lceil -90^{\circ },90^{\circ }\right\rceil ,\phi \in C,\left|\phi \right|=1\right\},$$

for a single snapshot of the received signal *A* under noise-free conditions z=A(θ)s, and the $l_{0}$-atomic norm is defined as follows:

(7) $${∥z∥}_{A,0}=\underset{s_{k},\theta_{k}}{\inf}\left\{K:z=\sum_{k=1}^{K}a\left(\theta_{k}\right)s_{k},\theta_{k}\in \left[-90^{\circ },90^{\circ }\right]\right\},$$

the $l_{0}$-atomic norm refers to the number of the smallest atoms that make up the vector z. In the context of DOA estimation, the problem is to find the combination of direction vectors with the smallest number of atoms. For a sparse array, the received signal z is often incomplete and imperfect. The process of signal recovery can be understood as filling the vector z in a way that minimizes the norm of the vector under the atom set *A*, i.e., finding the solution to

(8) $$\min_{z}{∥z∥}_{A,0}.$$

Next, using Lemma 1 of [28], for any positive semi-definite matrix $T\left(z\right)\in C^{L\times L}$, if r=rank(T(z))≤L−1, then the Hermitian Toeplitz matrix can be decomposed by a unique Vandermonde: $T\left(z\right)=\sum_{k=1}^{r}p_{k}a\left(\theta_{k}\right)a^{H}\left(\theta_{k}\right)=A\left(\theta \right)PA^{H}\left(\theta \right)$, where $A\in C^{L\times r}$ is a Vandermonde matrix whose columns are regarded as direction vectors for signals coming from different directions and $P=\mathrm{diag}\left(p\right)\in C^{r\times r}$ is a diagonal matrix. According to Lemma 2 of [28], we consider the following formula: $\left[\begin{array}{cc} x & z^{H}\\ z & T(z) \end{array}\right]\geq 0$, where x is a variable to be optimized. This formula corresponds to the following conclusion: (a) T(z)⪰0; (b) z must be located in the column vector of the Toeplitz matrix T(z). Following this, we modify the problem. Based on Conclusion (b) in Lemma 2, we ascertain that the signal z can always be linearly depicted by r sub-vectors in A(θ). These r sub-vectors are the r eigenvectors derived from the Vandermonde decomposition of the matrix T(z). Therefore, the problem of minimizing the $l_{0}$-atomic norm of vector z in (8) is equivalent to minimizing the rank of matrix (9), which can be expressed as

(9) $$\begin{array}{c} \min_{x,z}\mathrm{rank}\left(T\left(z\right)\right)\\ \;s.t.\;\left[\begin{array}{cc} x & z^{H}\\ z & T(z) \end{array}\right]⪰0 \end{array},$$

however, this optimization problem belongs to the class of non-deterministic polynomial (NP) problems, making it difficult to obtain an exact solution. Therefore, we relax the constraints in the original problem (8) to obtain a larger feasible region. Since the original problem seeks a minimum value, the optimal value of the relaxed problem must be less than or equal to the optimal value of the original problem. This enables the transformation of the original non-convex problem into a convex optimization problem. Similar to Equation (7), we define the $l_{1}$-atomic norm as

(10) $${∥z∥}_{A,1}=\underset{s_{k},\theta_{k}}{\inf}\left\{\sum_{k}\left|s_{k}\right|:z=\sum_{k=1}^{K}a\left(\theta_{k}\right)s_{k},\theta_{k}\in \left\lceil -90^{\circ },90^{\circ }\right\rceil \right\},$$

the original Problem (8) has been transformed into the following Problem (11) in form, which aims to recover a single snapshot signal vector z that minimizes its $l_{1}$-atomic norm. Problem (11) is represented as

(11) $$\min_{z}{∥z∥}_{A,1},$$

at this point, for ease of solution, we perform a Vandermonde decomposition on (11) again. After the decomposition, the problem can be transformed into Problem (12), which is represented as

(12) $$\begin{array}{c} \min_{x,z_{1}}\frac{1}{2}x+\frac{1}{2}z_{1}\\ \;s.t.\;\left[\begin{array}{cc} x & z^{H}\\ z & T(z) \end{array}\right]⪰0 \end{array},$$

where *x* is the variable to be optimized, $z_{1}$ denotes the first element of the single snap signal vector *z*, and T(z) is the Hermitian Toeplitz matrix with *z* as the first row. In the objective function, the first term $\frac{1}{2}x$ is a regular term set to prevent the appearance of the trivial solution, and the second term $\frac{1}{2}z_{1}$ is the trace norm of the T(z) matrix. Furthermore, the T(z) matrix is uniquely decomposed by the Vandermonde decomposition as

(13) $$T\left(z\right)=\sum_{k=1}^{r}p_{k}a\left(\theta_{k}\right)a^{H}\left(\theta_{k}\right),$$

after taking the trace of matrix (13), we obtain

(14) $$\mathrm{Tr}\left(T\left(z\right)\right)=L\sum_{k=1}^{r}p_{k},$$

in Equation (13), since the direction vectors $a(\theta_{k})$ are mutually independent, once we establish that the signal vector z exists in the vector space spanned by $a(\theta_{k})$, the coefficients $p_{k}$ can be uniquely determined. The lower-bound operation in thedefinition of the $l_{1}$ atomic norm in Equation (10) can be ignored, leading to the relationship between the $l_{1}$ atomic norm and the positive semidefinite matrix as

(15) $${∥z∥}_{A,1}=\mathrm{Tr}\left(T\left(z\right)\right)/L,$$

therefore, the convex Problem (12) is transformed into Problem (16), which is expressed as

(16) $$\begin{array}{cc} \; & \min_{z}\mathrm{Tr}\left(T\left(z\right)\right)\\ & s.t.\;T(z)⪰0 \end{array}.$$

Next, we can combine the virtual interpolation technique to solve the DOA estimation problem for augmented coprime array sensors. The procedure is as follows. Referring to Figure 4, we first calculate the covariance matrix $R_{X}$ based on the received signal information data from the actual antenna deployment in the set S. Then, we vectorize and sort the covariance matrix, removing redundant data. Here, we employ virtual interpolation techniques to replace the holes in the set D with zero elements, resulting in an equivalent single-snapshot signal vector $z_{ex}$ in the set V. Subsequently, using $z_{ex}$, we construct a reference virtual array covariance matrix $R_{V}$ containing the information from all array receive signal sources. Lastly, we define a binary matrix *G* of the same size as $R_{V}$ to reflect the positions where $R_{V}$ has non-zero values. By solving the optimization Problem (17) using an atomic norm minimization algorithm, we can obtain the reconstructed Toeplitz matrix T(z) containing the recovered single-snapshot signals.

(17) $$\begin{array}{c} \min_{z}\mathrm{Tr}\left(T\left(z\right)\right)\\ \;s.t.\;T(z)⪰0\\ {∥T\left(z\right)\circ G-R_{V}∥}_{F}^{2}\leq \eta \end{array},$$

where η is a threshold used to restrict the fitting error between the non-zero elements in $R_{V}$ and the non-zero values in the reconstructed covariance matrix T(z) projected onto *G*. The symbol ∘ denotes the Hadamard product, and ${\left|\cdot \right|}_{F}^{2}$ represents the squared Frobenius norm. For convenience of solving, the equation above can be reformulated as

(18) $$\begin{array}{cc} \min_{z} & \frac{1}{2}{∥T\left(z\right)\circ G-R_{V}∥}_{F}^{2}+\mu \mathrm{Tr}\left(T\left(z\right)\right)\\ \;s.t.\; & T(z)⪰0 \end{array},$$

where μ is a regularization parameter used to balance the fitting error and the atomic norm term.

### 3.3. Fast DOA Estimation via Positive Incremental Modified Cholesky Decomposition

When addressing the DOA problem via the atomic norm minimization method, one commonly encounters convex optimization challenges. Typically, these necessitate iterative optimization algorithms, such as interior-point methods. While these methods offer high accuracy, they also entail substantial computational complexity, particularly with large-scale problems, significantly slowing the solving process. For instance, employing an interior-point method to resolve a semidefinite programming optimization issue requires handling an N×N dimensional matrix, *T*, with a Hermitian Toeplitz structure. This operation demands $O(N^{3})$ matrix multiplications and inversions, yielding a computational complexity of $O(N^{3})$. Additionally, each iteration of the interior-point method entails solving an *N*-size linear system, with the iteration number typically being around O(logN). As a result, the total computational complexity escalates to $O(N^{3.5})$, limiting the method’s practical application. To enhance computational efficiency, we propose using the Cholesky decomposition algorithm. This algorithm pre-processes the coefficient matrix, which has a Hermitian Toeplitz structure, thereby reducing complexity and accelerating the semidefinite programming solution. Nevertheless, the conventional Cholesky decomposition method is suitable only for positive definite matrices, and our problem involves a positive semidefinite matrix. We thus propose a positive incremental modified Cholesky decomposition method based on the traditional model. Specifically, we add a very small positive value of $\delta =1\times 10^{-6}$ to the diagonal of the semipositive definite matrix, which is a common smaller value used for approximation in floating-point arithmetic. This operation also ensures that the elements on the diagonal are always positive, which avoids division-by-zero errors while minimising the impact on the matrix’s eigenvalues and turns the semipositive definite matrix into a positive definite matrix that can be processed using the Cholesky decomposition method [34]. For a given positive definite matrix *T*, its Cholesky decomposition is obtained as

(19) $$T=BB^{H},$$

where *B* is the lower triangular matrix of *T* and $B^{H}$ is the conjugate transpose of *B*. Now, we present the theoretical derivation process of the Cholesky decomposition algorithm to obtain the lower triangular matrix *B*. We assume the following:

(20) $$\begin{array}{c} B=\left(\begin{array}{c} \begin{array}{cccc} b_{11} & 0 & \cdots & 0\\ b_{21} & b_{22} & \cdots & 0\\ \vdots & \vdots & \ddots & \vdots \\ b_{n1} & b_{n2} & \cdots & b_{nn} \end{array} \end{array}\right)\\ B^{H}=\left(\begin{array}{c} \begin{array}{cccc} b_{11} & b_{21} & \cdots & b_{n1}\\ 0 & b_{22} & \cdots & b_{n2}\\ \vdots & \vdots & \ddots & \vdots \\ 0 & 0 & \cdots & b_{nn} \end{array} \end{array}\right) \end{array}.$$

Now, based on Equation (19), we seek the relationship between both sides of the equation. First, we can deduce $b_{11}=\sqrt{t_{11}}$ from $t_{11}=b_{11}^{2}$. Similarly, from $t_{i1}=b_{11}b_{i1}$, we obtain $b_{i1}=\frac{t_{i1}}{b_{11}}$, where i=2,3,...,n. This allows us to determine the elements of the first column of matrix *B*. Assuming that we have computed the first k−1 columns of matrix *B*, by performing the derivation using

(21) $$t_{kk}=\sum_{i=1}^{k}b_{ki}^{2},$$

we can obtain

(22) $$b_{kk}=\sqrt{t_{kk}-\sum_{i=1}^{k-1}b_{ki}^{2}},$$

furthermore, through the equation

(23) $$t_{ik}=\sum_{j=1}^{k-1}b_{ij}b_{kj}+b_{ik}b_{kk},$$

we can end up with

(24) $$b_{ik}=\frac{t_{ik}-\sum_{j=1}^{k-1}b_{ij}b_{kj}}{b_{kk}},$$

where i=k+1,…,n. This way, we can obtain the *k*th column of matrix *B* using the previously computed k−1 columns and continue this process iteratively to obtain the lower triangular matrix.

In practical applications, in order to speed up the solution of the gridless covariance matrix reconstruction problem based on ANM, we first use the positive incremental modified Cholesky decomposition method for the constraint matrix *T* of the original problem to obtain $T=BB^{H}$, followed by introducing a new transformation matrix *C*, as shown in the following Equation (25). This is applied to the solution of the optimization problem.

(25) $$T=B_{1}CB_{1}^{H},$$

where $B_{1}$ is a unit lower triangular matrix. Now, let us assume that *S* is a diagonal matrix containing the diagonal elements of matrix *B*. By combining Equations (19) and (25), we can deduce that $C=S^{2}$ and $B_{1}=BS^{-1}$. At this stage, we use the CVX [35] to determine the final covariance matrix by solving this newly transformed optimization problem as shown in Equation (26), which is a transformation of the original Problem (18).

(26) $$\begin{array}{cc} \min_{z} & \frac{1}{2}{∥C\left(z\right)\circ G-R_{V}∥}_{F}^{2}+\mu \mathrm{Tr}\left(C\left(z\right)\right)\\ \;s.t.\; & T=B_{1}C\left(z\right)B_{1}^{H} \end{array},$$

where C(z) denotes a Hermitian matrix with the vector z as its first column. By conducting a positive incremental modified Cholesky decomposition of the Toeplitz matrix, we simplify the original convex optimization problem. This transformation changes it into an equivalent optimization problem involving a transformation matrix, significantly reducing the computational complexity from $O(N^{3.5})$ to $O(N^{2.5})$. We derive the transformation matrix from the modified Cholesky decomposition of the Toeplitz matrix, a process with a computational complexity of $O(N^{2})$. Once decomposed, we use the transformation matrix to solve the equivalent optimization problem, which also requires a computational complexity of $O(N^{2})$. We employ the interior point method to solve the optimization problem. Each iteration necessitates solving a linear system iteration number problem carrying a computational complexity of $O(N^{0.5})$. As a result, the total computational expense becomes $O\left(N^{2.5}\right)$. Therefore, employing preprocessing based on the positive incrementally modified Cholesky decomposition proves beneficial for diminishing the computational cost of solving SDP problems involving symmetric Toeplitz matrices.

The reconstructed C(z) derived from Equation (26) aligns with a reference virtual ULA. Consequently, we can integrate various DOA estimation methods into the virtual domain for precise DOA estimation [36]. These methods encompass MUSIC-based, ESPRIT-based, and an array of sparsity-based techniques. Here, we employ the MUSIC method, which is represented by its spatial spectrum as

(27) $$f_{\mathrm{MUSIC}}\left(\theta \right)=\frac{1}{a^{H}\left(\theta \right)N_{C(z)}N_{C(z)}^{H}a\left(\theta \right)},$$

where $N_{C(z)}$ represents the noise subspace of C(z). Therefore, the estimation of the DOA can be obtained by performing a spectral peak search on the MUSIC method.

The proposed positive incremental modified Cholesky decomposition-based DOA estimation algorithm is summarized in Algorithm 1 and has the following key advantages. Initially, the method fully capitalizes on the non-uniform virtual array, denoted as D, by implementing the virtual array interpolation technique. This approach harnesses all available information in D, offering a robust foundation for more reliable DOA estimation. Next, the atomic norm minimization approach is used on equivalent virtual signals, allowing for the effective formulation of the optimization problem. The goal is to reconstruct the virtual array’s covariance matrix without depending on predefined grids. By avoiding grids, the common issue of grid mismatch is mitigated, thus improving the reconstruction process’s accuracy and robustness. Following this, a positive incremental modified Cholesky decomposition is applied to preprocess the reconstructed covariance matrix, T(z). This procedure, along with the introduction of a new transformation matrix, C(z), transforms the inequality constraints of the convex optimization problem into equality constraints. This improves the computational efficiency of the gridless approach and ensures reconstruction accuracy. Lastly, the MUSIC method is utilized to conduct statistical signal processing on the matrix C(z), which concludes in the final estimation of the arrival directions. It is worth noting that the PI-CANM algorithm proposed in this paper is not only applicable to reciprocal arrays, but it can also be applied to sparse arrays in which there are non-uniform virtual arrays in any virtual-domain signal processing, and its generalization to different arrays is relatively straightforward, differing only in the parameters *G* and $R_{V}$ related to the structure of non-uniform virtual arrays. In conclusion, the proposed algorithm demonstrates potential in augmented coprime array sensor DOA estimation. It theoretically improves DOA estimation accuracy and computational efficiency, with detailed simulation experiments to be presented in the following section.
**Algorithm 1** Positive incremental modified Cholesky decomposition-based DOA estimation.1:**Input:** Array receiving signal X(t).2:**Output:** $\theta =\left[\theta_{1},\theta_{2},\cdots ,\theta_{K}\right]$.3:**Initialize:** $R_{X},z_{ex},L$.4:Vectorize $R_{X}$, eliminate redundancy by sorting, and obtain the equivalent virtual signal $z_{ex}$ for the non-uniform virtual array D;5:Perform interpolation on the virtual array D to create a new virtual array V and use $z_{ex}$ to construct the Toeplitz reference matrix $R_{V}$ for V;6:Define a binary matrix *G* of the same size as $R_{V}$ to reflect the positions where $R_{V}$ has values;7:Perform a positive incremental modified Cholesky decomposition on the matrix T(z), which requires reconstruction, to obtain the transformation matrix C(z). Transform the convex optimization problem from Equation (18) to Equation (26);8:Solve the optimization problem constructed by Equation (26) to obtain the optimal solution C(z);9:Calculate the MUSIC spatial spectrum $f_{\mathrm{MUSIC}}$ using Equation (27) and estimate the directions of arrival by searching for the spectral peaks.

## 4. Simulation Results

In our simulations, we chose a pair of mutually prime integers M = 3 and N = 5 to deploy the augmented coprime array, which yielded a total number of 2M+N−1=10 physical sensors, positioned as the set S in Figure 4. We next analyzed the effectiveness of the proposed algorithm in terms of the following four aspects: degrees of freedom, resolution, estimation accuracy, and computational complexity.

### 4.1. Degrees of Freedom Analysis

In the field of array signal processing, degrees of freedom refer to the number of target directions that can be independently estimated, which is related to the number of sensors available in the array. In the first example, we assumed a set of far-field narrowband signal sources, covering 11 arrival angles ranging from −50 degrees to 50 degreeswith a 10-degree interval. The signal-to-noise ratio (SNR) was set to 0 dB, and the number of snapshots was L = 200. We compared the performance of the proposed algorithm with five coprime array sensor DOA estimation algorithms, including the SS-MUSIC algorithm [20], CO-LASSO algorithm [16], SBL algorithm [17], NNM algorithm [21], and ANM algorithm [28].

**Figure 5:**
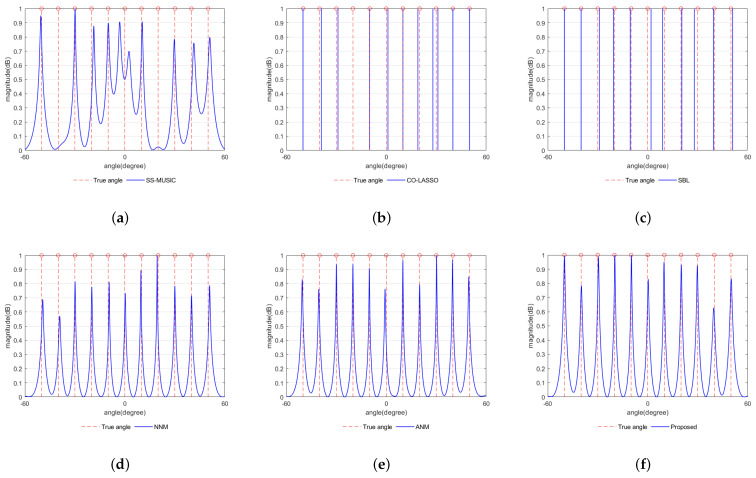
Resolution effect in terms of the normalized spatial spectrum with the number of snapshots L = 200. The vertical dashed lines denote the actual directions of the incident sources. (**a**) SS-MUSIC algorithm. (**b**) CO-LASSO algorithm. (**c**) SBL algorithm. (**d**) NNM algorithm. (**e**) ANM algorithm. (**f**) Proposed algorithm.

According to Figure 5, most algorithms can effectively resolve 11 signal sources using 10 physical elements. This demonstrates that DOA estimation using augmented coprime array sensors can increase the degrees of freedom. Further, the SS-MUSIC method did not use the discontinuous part of the virtual array element due to its ability to utilize only the continuous part of the virtual array element, which caused the loss of virtual aperture and degrees of freedom, resulting in the degradation of DOA estimation performance and large errors in DOA estimation for some sources. In addition, the CO-LASSO and SBL methods divide the continuous angle space into a set of discrete grids in advance and assume that the real angle is located between the grids, which inevitably results a base mismatch problem and cannot accurately estimate all signal sources. In contrast, the proposed method, along with the ANM and NNM methods, overcomes the limitations of grid-based approaches. It fills in the gaps in the virtual array and achieves higher accuracy and increased degrees of freedom compared to the other three methods. Therefore, the proposed method enables the accurate DOA estimation of 11 signal sources using only 10 elements.

### 4.2. Resolution Analysis

**Figure 6:**
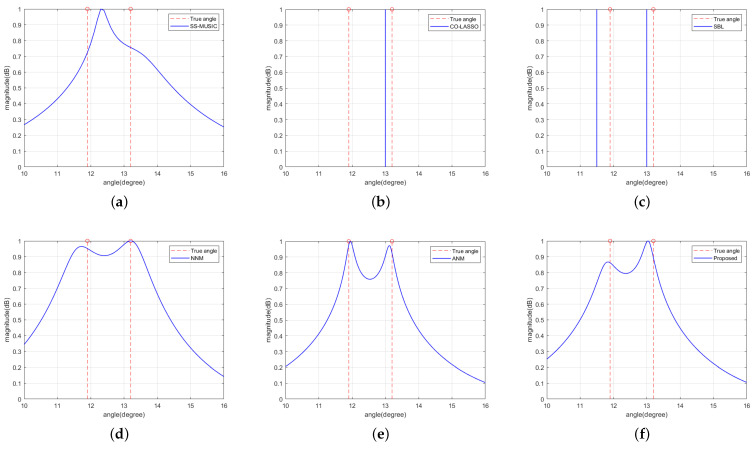
Resolution effect in terms of the normalized spatial spectrum with the number of snapshots L = 500. The vertical dashed lines denote the actual directions of the incident sources. (**a**) SS-MUSIC algorithm. (**b**) CO-LASSO algorithm. (**c**) SBL algorithm. (**d**) NNM algorithm. (**e**) ANM algorithm. (**f**) Proposed algorithm.

In the second example, we assumed there were two far-field narrowband signal sources with arrival angles of 11.9° and 13.2°, respectively. The SNR was set to 0 dB, and the number of snapshots was L = 500. Figure 6 shows the specific experimental results using the six methods from the first example under this experimental condition.

According to Figure 6, it can be seen that the SS-MUSIC method, CO-LASSO method, and SBL method cannot effectively resolve two signal sources with similar incident angles, while the proposed method has better resolution performance with the NNM and ANM methods. Therefore, under the same simulation conditions, the proposed method outperforms the SS-MUSIC method and CO-LASSO method in terms of resolution performance because the proposed method fills the cavity part of the non-uniform virtual array species by virtual interpolation to obtain more degrees of freedom and has a larger array element aperture, thus improving the resolution. Meanwhile, compared with the CO-LASSO and SBL methods, the proposed method does not require discretization of the angular domain and also effectively avoids the influence of the base mismatch problem on the resolution accuracy.

### 4.3. Estimation Accuracy Analysis

In the third example, we compared the estimation accuracy performance of the proposed method with several algorithms for DOA estimation using reciprocal arrays, including the SS-MUSIC algorithm, the CO-LASSO algorithm, the SBL algorithm, the NNM algorithm, and the ANM algorithm. We compared the root-mean-square-error (RMSE) of each algorithm in the example here to verify the effectiveness of the proposed algorithm, where the RMSE is defined as follows:

(28) $$\mathrm{RMSE}=\sqrt{\frac{1}{JK}\sum_{j=1}^{J}\sum_{k=1}^{K}{\left({\hat{\theta }}_{k}\left(j\right)-\theta_{k}\right)}^{2}},$$

where *J* represents the number of Monte Carlo trials, *K* denotes the number of signal sources, $\theta_{k}$ represents the true angle of the *k*-th incoming signal, and ${\hat{\theta }}_{k}\left(j\right)$ denotes the estimated value for the *k*-th angle in the *j*-th trial.

**Figure 7:**
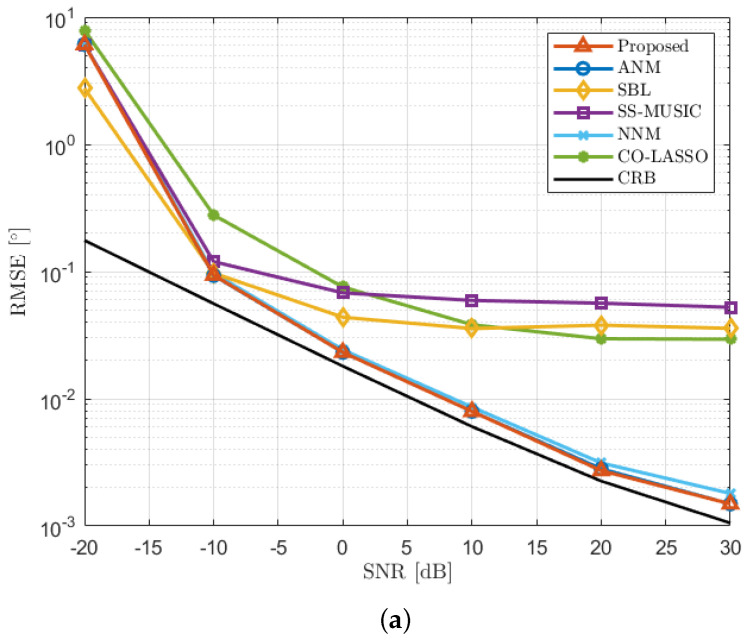

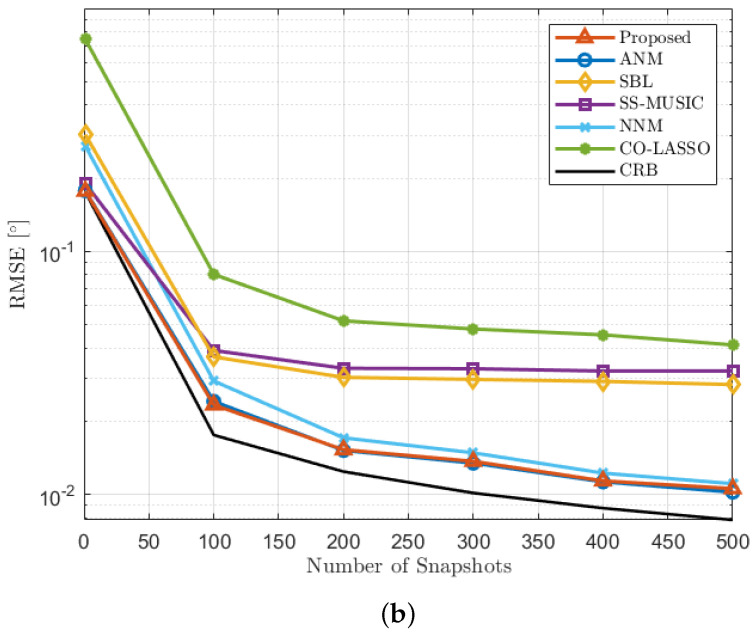
RMSE performance comparison with single incident source. (**a**) RMSE versus SNR with the number of snapshots T = 100. (**b**) RMSE versus the number of snapshots with SNR = 0 dB.

We assumed a single far-field narrowband signal source with an incoming azimuth angle following a Gaussian distribution with a mean of 5 degrees and a standard deviation of 2 degrees, denoted as $N\left(5^{\circ },2^{\circ }\right)$. This distribution was randomly generated in each Monte Carlo trial but remained unchanged within each sampling snapshot. The number of snapshots per trial was L = 100, and the total number of Monte Carlo trials was J=200. We conducted simulation experiments for several algorithms under uniformly varying SNR conditions ranging from −20 dB to 30 dB and observed the performance of the algorithms. Figure 7a illustrates the DOA estimation performance of each algorithm at different SNRs. From the figure, we can observe that the RMSE curve of the SS-MUSIC algorithm shows a flat trend when the SNR is greater than 10 dB. This is because the spatial smoothing operation in the algorithm can only act on the continuous part of the virtual array in the differential and array, which leads to a reduction in the array aperture, thus reducing the accuracy of DOA estimation. Similarly, the RMSE curves of the CO-LASSO and SBL algorithms also become relatively flat when the SNR exceeds 10 dB. This behavior is attributed to the requirement of predefined sampling grids in these sparse reconstruction algorithms, which leads to inherent basis mismatch and limits the estimation accuracy. In contrast, gridless algorithms such as NNM, ANM, and the proposed algorithm, do not require predefined sampling grids, and their estimation performance is not constrained by the sampling interval. Therefore, as the SNR increases, their RMSE curves exhibit a consistent variation trend with the Cramér–Rao bound (CRB) [37]. Notably, the CRB value is computed considering a single source, in line with the approach used for calculating the RMSE values for other algorithms. Among them, the performance of the proposed algorithm is almost the same as that of the ANM algorithm, and both are better than the performance of the NNM algorithm. Similar performance comparisons can also be found in Figure 7b, where the number of snapshots is different. The RMSE for the NNM algorithm is marginally higher compared to the proposed algorithm. This difference arises due to the NNM algorithm’s method of recovering the covariance matrix of the virtual interpolated array based on a matrix completion criterion. This process involves retaining a select number of observation values computed from sampled snapshots within the optimized covariance matrix. As a result, the recovered covariance matrix produced by the NNM algorithm might contain certain inaccuracies. Conversely, the proposed algorithm employs these relevant observation values only as reference points, and it reconstructs the covariance matrix of the virtual interpolated array following a matrix reconstruction criterion. Consequently, the proposed algorithm exhibits superior performance compared to the NNM algorithm.

### 4.4. Computational Complexity Analysis

The computational complexity of the algorithm is also one of the indicators for evaluating the performance of the algorithm, so in the fourth example, we used the same six algorithms used in the third example to conduct simulation experiments. We set them under the condition of different number of array sensors, completed DOA estimation, and recorded the time required. The experiments were conducted in the MATLAB 2022 platform using an Intel Core i5-10400 processor (Intel, Santa Clara, CA, USA) and 16 GB of memory. Here, we assumed there was one signal source, the snapshot count was L = 10, the SNR = 0 dB, and the number of array sensors varied from 7 to 37.

**Figure 8:**
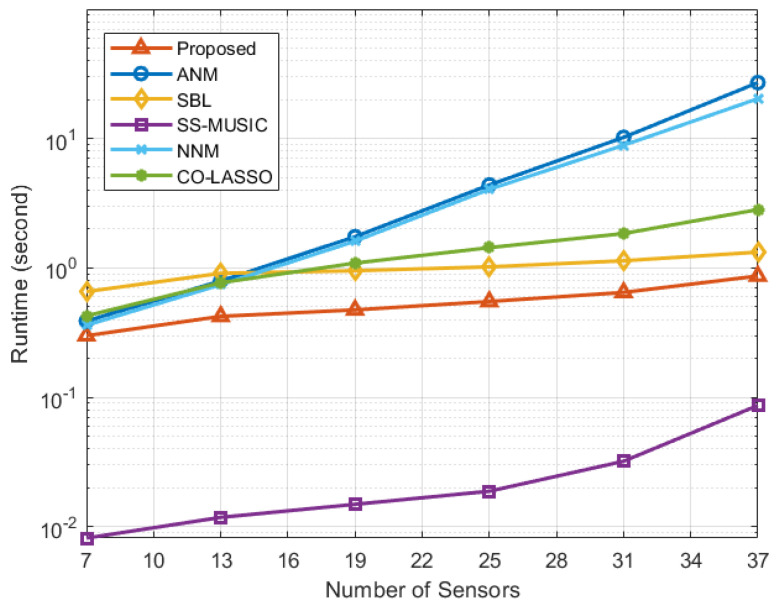
Runtime performance versus number of sensors.

Figure 8 evaluates the computational cost of various algorithms by comparing their runtimes under different numbers of array sensors, N. We observe that although the calculation speed of the SS-MUSIC algorithm is faster than the proposed algorithm, its RMSE performance is relatively poor. In scenarios where precision of DOA estimation is required, this degradation in performance cannot be overlooked. Furthermore, compared to the proposed algorithm, both the SBL and CO-LASSO algorithms are slower in computation, as they require more computational resources and iterations to address intricate optimization problems. Notably, among the three gridless algorithms, our proposed algorithm exhibits the fastest calculation speed. This is attributed to the implementation of modified Cholesky decomposition during the solution of convex optimization problems in matrix recovery. It transforms the matrix from semi-definite positive to definite positive, simplifying the constraints of the optimization problem and thus reducing the complexity of the solution. Furthermore, as N increases, the slope of the runtime curve for our proposed algorithm becomes notably smaller than that of the other two gridless algorithms, reflecting a lower order of computational complexity in an asymptotic sense. In conclusion, while ensuring the accuracy of DOA estimation, the proposed algorithm is more suitable for large-scale problems and scenarios demanding high real-time algorithmic performance.

## 5. Conclusions

We introduce a PI-CANM-based DOA estimation algorithm for augmented coprime array sensors. This approach addresses information loss caused by the discontinuity of the virtual array through virtual interpolation array element technology. It reformulates the DOA estimation issue as a gridless optimization problem, circumventing limitations of pre-defined spatial grid points on DOA accuracy. It employs the positive incremental modified Cholesky decomposition method to ease the optimization problem’s constraints and reduce complexity. Simulation comparisons confirm the algorithm’s robustness, even in conditions such as low signal-to-noise ratios and small snapshots, and highlight its practical applicability. We consider this a valuable DOA estimation method for coprime array sensors that will be useful for related research fields. 

## Data Availability

Not applicable.
